# Surveillance of Mosquitoes and Selected Arthropod-Borne Viruses in the Context of Milan EXPO 2015

**DOI:** 10.3390/ijerph13070689

**Published:** 2016-07-08

**Authors:** Mario Chiari, Mattia Calzolari, Alice Prosperi, Simona Perulli, Francesca Faccin, Dominga Avisani, Monica Cerioli, Mariagrazia Zanoni, Marco Tironi, Marco Bertoletti, Francesco Defilippo, Ana Moreno, Marco Farioli, Alessandra Piatti, Michele Dottori, Davide Lelli, Antonio Lavazza

**Affiliations:** 1 Istituto Zooprofilattico Sperimentale della Lombardia e dell’Emilia Romagna “Bruno Ubertini”, Brescia 25124, Italy; mattia.calzolari@izsler.it (M.C.); alice.prosperi@izsler.it (A.P.); simona.perulli@izsler.it (S.P.); francesca.faccin@izsler.it (F.F.); dominga.avisani@izsler.it (D.A.); monicapierangela.cerioli@izsler.it (M.C.); mariagrazia.zanoni@izsler.it (M.Z.); marco.tironi@izsler.it (M.T.); marco.bertoletti90@gmail.com (M.B.); francesco.defilippo@izsler.it (F.D.); anamaria.morenomartin@izsler.it (A.M.); michele.dottori@izsler.it (M.D.); davide.lelli@izsler.it (D.L.); antonio.lavazza@izsler.it (A.L.); 2 Regional Health Authority of Lombardy, Milan 20124, Italy; Marco_farioli@regione.lombardia.it (M.F.); Alessandra_piatti@regione.lombardia.it (A.P.)

**Keywords:** EXPO 2015, entomological surveillance, mosquito-borne diseases, Chikungunya, Dengue, West Nile virus, Lombardy region

## Abstract

From 1 May 2015 to 31 October 2015 over 20 million visitors from all over the world visited the Universal Exhibition (EXPO) hosted by Milan (Lombardy region, Italy), raising concerns about the possible introduction of mosquito-borne diseases from endemic countries. The entomological surveillance protocol performed in Lombardy over the last three years was implemented in the EXPO area and in the two major regional airports using both Center for Disease Control CO_2_ and Biogents Sentinel traps. This surveillance aimed to estimate the presence and densities of putative vectors, and also to support investigations, including the vector species involved and area of diffusion, on the local spread of Chikungunya, Dengue and West Nile viruses (WNV) by competent vectors. From 3544 mosquitoes belonging to five different species, 28 pools of *Culex* spp. and 45 pools of *Aedes* spp. were screened for the presence of WNV, and for both Chikungunya and flaviviruses, respectively. The entomological surveillance highlighted a low density of potential vectors in the surveyed areas and did not reveal the presence of Chikungunya or Dengue viruses in the local competent vectors inside the EXPO area or in the two airports. In addition, the surveillance reported a low density of *Culex* spp. mosquitoes, which all tested negative for WNV.

## 1. Introduction

Mass gathering events can be associated with outbreaks of communicable and/or exotic diseases because of the crowded conditions within a confined area and the close contact between participants. In particular, the possibility of introducing mosquito-borne diseases (MBDs) was considered as an important health risk given the proven capability of competent mosquitoes to promote the local spread of diseases introduced into temperate regions [1,2].

Milan (Lombardy region, Italy) hosted the Universal Exhibition (EXPO) from 1 May 2015 to 31 October 2015, showcasing exhibits from more than 140 participating countries. Over 20 million visitors from all over the world visited the 1.1 million square meters of exhibition area. Because of the reasons mentioned above, as well as the influx of equipment and people, most of whom were from non-European countries, into the EXPO area, concerns were raised about the emergence or re-emergence of diseases. 

The risk of a local emergence of an MBD is real because the exotic mosquito *Aedes albopictus* (Skuse, 1894), a competent vector for Dengue virus (DENV) and Chikungunya virus (CHIKV), is well established in northern Italy [3]. This was demonstrated by the Chikungunya disease outbreak in 2007 in Castiglione, which resulting in more than 200 human cases [4]. In addition, *Culex pipiens* (Linnaeus, 1758), a competent vector for the West Nile virus (WNV), has played a major role in the transmission of the virus in recent outbreaks in Lombardy, as well as in the neighboring Emilia-Romagna, Veneto and Piedmont regions [5], where WNV can be considered endemic. For these reasons, as well as the movements of travelers and trades in the context of the EXPO, the potential risk of an MBD emerging existed. Based on the European human cases of DENV- and CHIKV-associated diseases from 2002 to 2012, short-term travelers (mainly tourists and business travelers) were considered the highest risk group for carrying such diseases [6]. In addition, the threat of the possible introduction and potential establishment of CHIKV and DENV during the EXPO was increased by the exhibition period (1 May to 31 October) coinciding with the vectors’ season and the favorable transmission period of MBDs in Italy, as well as in other European countries [6]. It should be emphasized that this summer period also coincided with the transmission period of WNV in northern Italy, where it is considered endemic [5]. From 2008 to 2014, this virus was identified in vectors, birds and horses in northern Italy, including the Lombardy region [5]. Thus, entomological and ornithological surveillance was modified and strengthened year after year to ensure a more accurate surveillance system. Considering the epidemiological complexities of MBDs, in which vectors, pathogens and hosts interact under the strong influence of environmental conditions, active vector surveillance is necessary for MBD identification and control [7]. It is essential to not only evaluate the presence and densities of the vectors, but also the possible presence and prevalence of the pathogens in the vector population, as well as the intensity of virus transmission in an area.

Events that move millions of visitors from all over the world to a specific area for a short period of time (such as the EXPO or the Olympic Games) result in a high risk of MBD introduction [6]. During these kinds of events, active vector surveillance is necessary for the evaluation of the presence and densities of competent vectors, and the possible early identification of MBDs in resident mosquitoes. This study describes the entomological surveillance performed in the context of the EXPO to estimate the vectors’ presence and densities, and to identify possible agents of MBDs in resident mosquitoes. These surveillance activities were implemented in light of the potential risks of emerging MBDs, caused by DENV and CHIKV, due to the influx of international travelers and trades to the EXPO event. In addition, this surveillance could detect locally endemic MBD agents, such as WNV, in competent vectors, with the aim of implementing preventive measures to protect EXPO visitors.

## 2. Materials and Methods

The entomological surveillance performed in Lombardy since 2014 [8] was implemented and integrated into a specific surveillance system performed from the beginning of June to the end of September 2015. This surveillance was used inside the EXPO area and in the two major airports of the Lombardy region, Malpensa International Airport and Linate City Airport, because the people and equipment involved in the EXPO were expected to transit mainly through these airports. Specific vector-control activities were not implemented in the airport areas, but were organized and intensified during the summertime at the EXPO site, with the repeated adoption of both larvicidal and adulticidal treatments, particularly focused on potential larval breading sites and the network of green areas.

Because the use of accurate mosquito-trapping methods is crucial in vector surveillance, two different kinds of traps were used to obtain a comprehensive sampling (Table 1). Thus, mosquito sampling involved the collection of insects at the three sites using two different attractive traps per site, modified Center for Disease Control (CDC) CO_2_ dry-ice baited traps [9] and Biogents (BG) Sentinel traps with BG-Lure attractant (Biogents, Regensburg, Germany). The two traps were placed outdoors with a distance of 150 m between them at each site. CDC-CO_2_ traps were suspended 1.5–2 m from the ground and worked for one night, from 5 p.m. to 9 a.m. the next day, while the BG Sentinel traps were placed on the ground and worked for a 24 h period. To limit the bias due to the sampling activities, the positions of the traps inside the selected areas had been chosen to maximize the number of captured mosquitoes. The traps inside the EXPO were placed in the green areas, in the proximity of small trees between two small ditches, which had a constant low water level. Inside the airports, traps were placed near hedges, in shaded, quiet areas with low levels of vehicular movement. Both of the traps (CDC-CO_2_ and BG) in each site worked on the same night. Fortnightly capture sessions in which mosquitoes were trapped every 14 days, for a total of eight trapping nights per site, were carried out by local veterinary authorities in the interest of collecting the greatest number and variety of host-seeking females [10]. Trapped insects were maintained alive in the original sampling bag and kept refrigerated (4 °C) to preserve the viruses potentially present from degradation during delivery to the laboratory. At the Istituto Zooprofilattico Sperimentale della Lombardia e dell’Emilia Romagna, mosquitoes were identified at the species level using morphological keys [10,11]. The analyses to detect the WNV genome were carried out only on mosquitoes belonging to the *Culex* genus, as these mosquitoes are considered a highly competent vector for WNV in the study area, as well as the other regions in northern Italy [2,8]. The presence of DENV and CHIKV was assessed in *Ae. albopictus,* the main competent vector for these viruses in the study area [12], and also in *Aedes (Ochlerotatus) caspius* (Pallas, 1771), which does not seem refractory to CHIKV infections under laboratory conditions [13].

Mosquitoes were divided into pools based on species, date and trapping location, with a maximum of 100 specimens per pool. Pools were homogenized in phosphate-buffered saline in a 2 mL microtube with copper-plated beads and then kept at −80 °C until viral screening. Total RNA was extracted from mosquito samples according to the Qiazol^®^ (Qiagen, Hilden, Germany) manufacturer’s instructions. Pools of the *Culex* genus were tested using a QuantiFast Pathogen RT-PCR +IC Kit (Qiagen, Hilden, Germany) for WNV, according to Tang et al. [14]. *Aedes* spp. samples were tested using a pan-flavivirus hemi-nested One-Step RT-PCR protocol to detect DENV, as previously described by Scaramozzino et al. [15]. This test could allow the detection of other flaviviruses of extreme importance, such as Zika or Yellow fever [15]. At the same time a TaqMan^®^ One-Step RT-PCR protocol, as described by Pastorino et al. [16], was used to detect CHIKV.

## 3. Results and Discussion

A total of 3544 mosquitoes belonging to five different species were collected, and *Cx. pipiens* (52.7%) and *Ae. caspius* (45%) represented the most common species trapped, while other species were detected at lower abundances and with more scattered distributions (Table 1), as already described in the Lombardy region [8].

The CDC-CO_2_ traps were more effective in collecting *Cx. pipiens* and *Ae. Caspius*, principally because of the carbon dioxide used as an attractant, as previously stated [9]. The total number of mosquitoes sampled indicated their low abundance when compared with previous data from the same region [8]. This could be the effect of the specific vector-control activities implemented during the summertime at the EXPO site and at the public green area surrounding Linate Airport. Even at a low density, *Cx. pipiens*, the main vector of WNV in northern Italy [5], was regularly collected inside the EXPO site and from the Linate Airport area. The mosquito samples collected from theMalpensa Airport area were different when compared with those from the other two sites. In fact, no specimens of *Ae. albopictus* were collected at Malpensa Airport, while a great number of *Ae. (Ochlerotatus) caspius* were sampled. The low density of mosquitoes sampled inside the airports could be due to these areas having less favorable environmental conditions for the presence and development of stable and consistent mosquito populations. This is true, in particular, for *Ae. albopictus* and *Cx. pipiens*. The urban environment is considered much more favorable to the population development of *Ae. albopictus*, and, when suitable loci for *Cx. pipiens* larvae growth exist, this species can also reach a high density [10]. In contrast, the urban areas are not favorable to *Ae. caspius* populations [17,18]. This species was already described as an aggressive human-biting mosquito in northern Italy [17], and it may act as a potential bridge vector for arboviruses [13,18]. The location of Malpensa Airport, surrounded by both cultivated fields and woodlands, and in close proximity to the Natural Park of Ticino Valley, influenced the mosquito population. Thus, the sampling results were directly tied to the site characteristics and, even though two sites were inside airports, the natural surrounding areas also influenced the vector distribution.

The number of trapped mosquitoes reflected a low density of vectors in the sampled areas. In total, 28 pools of *Cx. pipiens*, 10 of *Ae. albopictus* and 35 of *Ae. caspius* from the three monitored sites were virologically tested, without any positive results for the presence of the target viruses. This result is consistent with the Regional Health Authorities data, which confirmed that none of the 20 DENV-associated disease cases in humans reported in Milan Province could be directly linked to EXPO activities. These were all imported cases: specifically, 13 acquired in Asia, six in central South America and one in Africa. The number of cases of DENV-associated diseases in humans reported in 2015 was similar to the numbers recorded over the last three years in the region (25 cases in 2012; 42 cases in 2013; 14 cases in 2014). For each case, the measures foreseen by the Ministry of Health on the surveillance of vector-borne diseases were adopted by the Regional Health Authorities, including adulticidal treatments in areas surrounding the domiciles of infected people.

**Figure 1:**
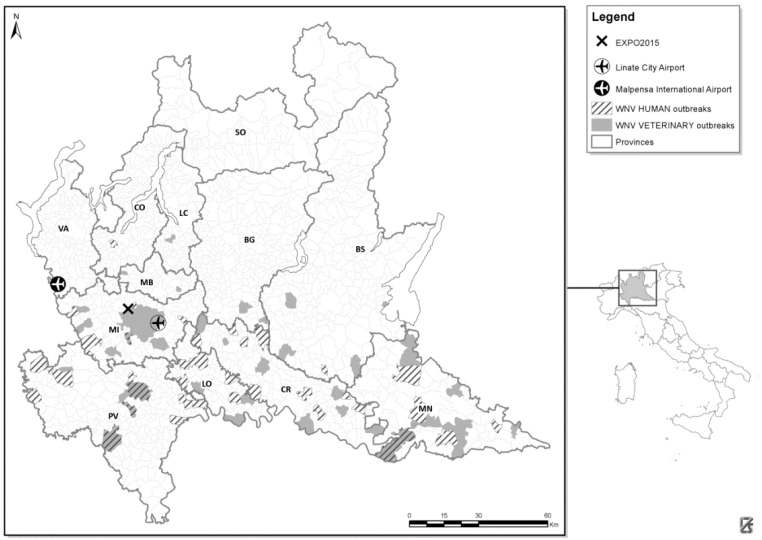
Areas of West Nile virus circulation in 2014 and 2015 in the Lombardy region reporting positive human (clinical case) and veterinarian (vectors, birds and horses) cases during surveillance activities [19].

Although none of the 28 pools of *Culex* spp. were positive for WNV, the WND Regional surveillance plan confirmed the circulation of WNV during the summer of 2015 in several Lombardy areas (Figure 1) [19]. 

WNV transmission is characterized by a complex epidemiological cycle, depending particularly on specific ecological conditions, such as the richness and abundance of mosquitoes and birds [20]. The lack of WNV detection in the EXPO area could be due to the absence of the specific environmental conditions that are required for the establishment of the WND transmission cycle. Even if WNV-competent vectors were present, the absence of the required abundance and richness of WNV reservoirs, probably due to the recent urbanization of the EXPO area, could be the cause of the absence of the circulating virus. Similarly, in the two monitored airports, where strict bird management procedures are routinely carried out to prevent wildlife-aircraft collisions, the ecological conditions were likely not appropriated for the establishment of the WNV transmission cycle. The bird management protocol includes species, such as magpie (*Pica pica*), hooded crow (*Corvus corone cornix*) and Eurasian jays (*Garrulus glandarius*), that are among the major reservoirs of WNV.

An added value of this surveillance could be the use of a pan-flavivirus hemi-nested RT-PCR protocol that can detect other flaviviruses, such as the recently emerged Zika virus. In fact, from a pool of *Ae. (Ochlerotatus) caspius* collected inside the Malpensa Airport in mid-July, a flavivirus strictly related to the Marisma virus [21], most likely a non-pathogen recently found in the neighboring region [22], was detected.

## 4. Conclusions

Prerequisites for the possible autochthonous transmission of an MBD (caused by WNV, DENV or CHIKV), including the presence of competent vectors and suitable climatic conditions, were present in the monitored areas. Nevertheless, our study demonstrated a low density of the main competent vector, *Ae. albopictus*, inside the EXPO area and in the two major airports of the Lombardy region, and excluded the presence of the selected MBDs in all of the insect pools examined.

Entomological surveillance for the introduction of new MBDs should be implemented not only in the event area, but also in the main airports used by visitors. The imported cases of MBDs through international travel may be the consequence of the infection of local vector populations by new hosts (i.e., infected travelers), or it may be due to the introduction of mosquitoes carrying a virus into a new environment [23]. 

To better estimate the risk of MBD introduction and diffusion, entomological surveillance and other surveillance strategies, such as the accurate clinical surveillance of humans in the area, based on hospital admissions, and the development of case definitions of the targeted diseases using laboratory diagnostics, should be maintained during and even after any international event, such as the EXPO, to enable the detection of a possible silent infection and, possibly, before an uncontrollable expansion of the infection.

## Figures and Tables

**Table 1:** Summary of the number and species of mosquitoes trapped during the surveillance activities in the EXPO framework. Fortnightly night-trap sessions were carried out in each of the three selected sites using BG Sentinel traps with BG-Lure attractant and CDC-CO_2_ dry ice-baited traps.

Species of Mosquitoes	EXPO Area		Linate Airport		Malpensa Airport		Total
	BG Sentinel	CDC-CO_2_	BG Sentinel	CDC-CO_2_	BG Sentinel	CDC-CO_2_	
*Cx. pipiens*	153	784	11	912	0	7	1867
*Cx. modestus*	0	0	0	1	0	0	1
*Ae. albopictus*	23	2	6	18	0	0	49
*Ae. caspius*	8	53	0	47	4	1482	1594
*An. maculipennis*	4	21	0	4	0	4	33
Total	188	860	17	982	4	1493	3544

## Abbreviations

The following abbreviations are used in this manuscript:

EXPO 2015	Universal Exhibition 2015
MBDs	Mosquito-borne diseases
DENV	Dengue virus
CHIKV	Chikungunya virus
WNV	West Nile virus
CDC	Centre for Disease Control
BG	Biogents
